# Systematic evaluation of agarose- and agar-based bioinks for extrusion-based bioprinting of enzymatically active hydrogels

**DOI:** 10.3389/fbioe.2022.928878

**Published:** 2022-11-21

**Authors:** Lukas Wenger, Carsten P. Radtke, Eva Gerisch, Max Kollmann, Christof M. Niemeyer, Kersten S. Rabe, Jürgen Hubbuch

**Affiliations:** ^1^ Institute of Engineering in Life Sciences, Section IV: Biomolecular Separation Engineering, Karlsruhe Institute of Technology, Karlsruhe, Germany; ^2^ Institute for Biological Interfaces 1, Karlsruhe Institute of Technology, Karlsruhe, Germany

**Keywords:** 3D printing, bioprinting, enzymes, esterase, biocatalytic reactors, enzyme leaching, enzyme activity

## Abstract

Extrusion-based 3D bioprinting enables the production of customized hydrogel structures that can be employed in flow reactors when printing with enzyme-containing inks. The present study compares inks based on either low-melt agarose or agar at different concentrations (3–6%) and loaded with the thermostable enzyme esterase 2 from the thermophilic organism *Alicyclobacillus acidocaldarius* (AaEst2) with regard to their suitability for the fabrication of such enzymatically active hydrogels. A customized printer setup including a heatable nozzle and a cooled substrate was established to allow for clean and reproducible prints. The inks and printed hydrogel samples were characterized using rheological measurements and compression tests. All inks were found to be sufficiently printable to create lattices without overhangs, but printing quality was strongly enhanced at 4.5% polymer or more. The produced hydrogels were characterized regarding mechanical strength and diffusibility. For both properties, a strong correlation with polymer concentration was observed with highly concentrated hydrogels being more stable and less diffusible. Agar hydrogels were found to be more stable and show higher diffusion rates than comparable agarose hydrogels. Enzyme leaching was identified as a major drawback of agar hydrogels, while hardly any leaching from agarose hydrogels was detected. The poor ability of agar hydrogels to permanently immobilize enzymes indicates their limited suitability for their employment in perfused biocatalytic reactors. Batch-based activity assays showed that the enzymatic activity of agar hydrogels was roughly twice as high as the activity of agarose hydrogels which was mostly attributed to the increased amount of enzyme leaching. Agarose bioinks with at least 4.5% polymer were identified as the most suitable of the investigated inks for the printing of biocatalytic reactors with AaEst2. Drawbacks of these inks are limited mechanical and thermal stability, not allowing the operation of a reactor at the optimum temperature of AaEst2 which is above the melting point of the employed low-melt agarose.

## 1 Introduction

Additive manufacturing (AM) — or 3D printing — is a dynamically evolving field offering versatile and highly adaptive fabrication methods that are useful for a wide range of applications (Ngo et al., 2018). It is based on the stacking of layers to gradually build three-dimensional objects. While initially mostly applied for visualization models and rapid prototyping, the advancement of printing technologies and materials has made 3D printing a more commonly applied method for the fabrication of working prototypes and even functional parts for end use (Gibson et al., 2021). This can be mainly attributed to the availability of AM methods allowing the fabrication of parts with excellent mechanical stability, e.g., made from metal (Blachowicz et al., 2021) or ceramics (Chen et al., 2019), that can be employed in demanding applications. Many fields, ranging from the aerospace (Joshi and Sheikh, 2015), automotive (Leal et al., 2017) and construction (Wu et al., 2016) industry to the chemical engineering (Parra-Cabrera et al., 2018) and biotechnology (Krujatz et al., 2017) sector, are investigating how to exploit the new possibilities provided by 3D printing. In (bio-)chemical engineering, two general fields can be distinguished based on the employed types of material. On one side, conventional materials like glass (Gal-Or et al., 2019), metal (Gupta et al., 2016) or water-free polymers (Simon et al., 2020) are used in the production of microfluidic devices or chromatography columns. On the other side, soft and biocompatible, water-based materials like hydrogels are employed with the purpose of accommodating cells (Billiet et al., 2014; Tabriz et al., 2015) or biomolecules (Mandon et al., 2016; Devillard et al., 2018) in a suitable aqueous environment.

This discipline, often referred to as *bioprinting* as a branch of *biofabrication* (Groll et al., 2016), is mainly focused on the development of new methods, tools and materials for tissue engineering (Ozbolat and Hospodiuk, 2016). Recently, those tools were increasingly adopted for the fabrication of biocatalytic flow reactors based on cells (Saha et al., 2018) or enzymes (Maier et al., 2018; Schmieg et al., 2018; Peng et al., 2019; Wenger et al., 2020). Such reaction systems, both on a microfluidic and macroscopic scale, play an important role in biocatalytic applications like compartmentalized catalytic cascades which can be realized by employing spatially separated reaction chambers (Rabe et al., 2017).

Hydrogels can serve as a matrix for the immobilization of enzymes by physical entrapment, thereby avoiding the loss of the catalyst and enhancing cost-efficiency (Krishnamoorthi et al., 2015). This method offers a simple way of immobilization, usually without the need to adapt it to a specific enzyme. The immobilization of enzymes in hydrogels is based on the entrapment of the relatively large enzyme within a polymer network while allowing small substrate and product molecules to diffuse in and out of the hydrogel (Nisha et al., 2012). Choosing a support material with an appropriate pore size is required to maximize the diffusion of substrate and product while minimizing the leaching of enzyme (Górecka and Jastrzȩbska, 2011). A drawback of the method is limited mass transfer of substrate and product and hence decreased activity (Nisha et al., 2012; Schmieg et al., 2018). In order to compensate for this limitation, it is paramount to maximize the surface-area-to-volume ratio of the hydrogel (Wenger et al., 2020).

Bioprinting enables the rapid and flexible fabrication of appropriate hydrogel structures with a high surface-area-to-volume ratio and perfusable geometries like simple grids (Maier et al., 2018; Peng et al., 2019) or more complex gyroid structures (Wenger et al., 2020) that can be employed in microreactors. To achieve high surface-area-to-volume ratios and complex geometric shapes, it is paramount to optimize the printability of the employed materials. Unlike molding methods, bioprinting allows quick adaptations to new geometries without the need to fabricate new molds and enables the fabrication of certain geometries like gyroid structures that are not manufacturable using common molding techniques. Spatially separated or compartmentalized enzymatic reactions can be realized by employing multiple print heads that allow the deposition of different enzymes in a spatially controlled manner. Overall, the flexible and fast fabrication of biocatalytically active constructs by additive manufacturing can accelerate iterative optimization processes and contribute to the advancement of the field (Rabe et al., 2017).

One of the most common techniques in biofabrication is extrusion-based bioprinting, a simple method based on the deposition of fluid materials from a cartridge through a nozzle onto a substrate (Panwar and Tan, 2016). It requires the use of (bio-)inks that can be printed in a fluid state and be solidified after deposition, forming stable hydrogels. Suitable rheological properties like a high viscosity or high yield stress (Mouser et al., 2016) are vital factors to ensure good printability (Hölzl et al., 2016). Typically, water-soluble polymers like gelatin (Yin et al., 2018), hyaluronic acid (Pescosolido et al., 2011) or alginate (Tabriz et al., 2015; Axpe and Oyen, 2016) are used, often accompanied by additives like methyl cellulose (Contessi Negrini et al., 2018; Law et al., 2018) or nanosilicates (Wilson et al., 2017; Peak et al., 2018) to enhance and adapt rheological properties.

Unlike cells, enzymes do not require any nutrient supply and can often handle harsher environments, opening up new options regarding bioprinting methods and materials. Current approaches include the use of poly(ethylene glycol) diacrylate-based hydrogels (Schmieg et al., 2018) or emulsion-based hybrid materials (Wenger et al., 2020) to print catalytically active materials containing β-galactosidase. Both these approaches rely on UV curing, introducing the potential of enzyme inactivation due to the presence of free radicals. As an alternative material system based on natural polymers, agarose and agar hydrogels are potential candidates. Agarose and agar can be dissolved in water at elevated temperature and form stable hydrogels upon cooling (Renn, 1984; Sassolas et al., 2013) which makes them interesting materials to be employed in bioprinting. Agarose is a naturally occuring polymer that is the main component of agar which can be extracted from red algae (*Rhodophyceae*) (Armisén, 1991).

**FIGURE 1:**
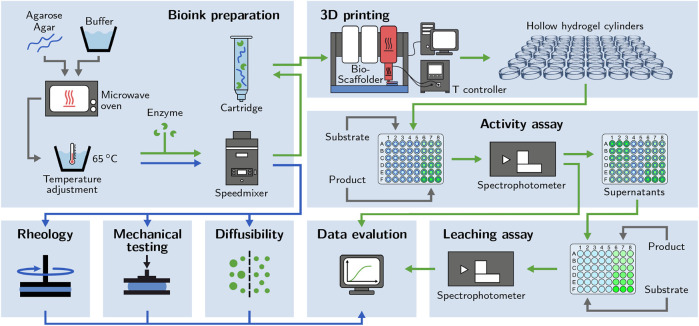
Schematic of the applied workflow. Bioinks based on different concentrations of low-melt agarose and agar are prepared. Enzyme-loaded hydrogel cylinders are printed to perform activity assays. The supernatants are used for leaching assays. Enzyme-free bioinks and hydrogels are examined with rheological analysis, mechanical testing and diffusibility measurements.

We have shown before that hydrogels based on modified low-melt hydroxyethyl agarose are principally applicable for the printing of thermostable enzymes (Maier et al., 2018; Peng et al., 2019). However, no optimization of the printing setup and procedure and no systematic screening of different bioink compositions has been performed yet. In the published studies, an arbitrarily chosen and fixed hydrogel composition of 3% (w/v) low-melt hydroxyethyl agarose was used to print modules for perfusable biocatalytic reactors (Maier et al., 2018; Peng et al., 2019). The printing setup was not specifically optimized for the printing of thermosensitive materials which resulted in the extrusion of agarose hydrogels in a partially gelled state causing low printing quality and irregular prints. The present study shows the further development of the printing setup by introducing a heatable nozzle that allows for a more precise control of the bioink temperature upon extrusion to achieve more reproducible and robust prints. Furthermore, we assess a range of bioinks prepared from two different materials (low-melt hydroxyethyl agarose and unmodified agar) at different concentrations [3–6% (w/w)] to explore the potential for optimizing printability and catalytic activity by changing the ink compositions. The liquid inks are assessed for rheological properties and gelation behavior. The solidified hydrogels are investigated regarding mechanical stability and diffusibility. Enzyme-containing hydrogel samples are printed and analyzed for enzyme leaching and biocatalytic activity using microplate-based batch activity assays. The screening of a range of inks with slightly different compositions with regard to multiple aspects demonstrates the importance of adapting inks specifically for certain applications. A schematic overview of the applied workflow of the study is presented in Figure 1.

## 2 Materials and methods

### 2.1 Chemicals and buffers

Agar (bacteriology grade) was purchased from AppliChem GmbH, low-melt agarose (Roti^®^garose with low melting and gelling temperature) from Carl Roth GmbH & Co. KG. All hydrogels were prepared with phosphate buffered saline (PBS), pH 7.4. Sodium chloride (NaCl), potassium chloride (KCl), disodium hydrogen phosphate dihydrate (Na_2_HPO_4_ ⋅ 2 H_2_O) and potassium dihydrogenphosphate (KH_2_PO_4_) were purchased from Merck KGaA. The PBS buffers were prepared with ultrapure water from a Purelab Ultra water purification system (ELGA LabWater) and filtered through an 0.2 µm cellulose acetate filter (Sartorius AG) before use. 5(6)-carboxyfluorescein dihexylester was synthesized at the Institute for Biological Interfaces 1 and used as a substrate for activity assays.

### 2.2 Enzyme expression and purification

As a model enzyme, esterase 2 from the thermophilic organism *Alicyclobacillus acidocaldarius* (AaEst2, EC 3.1.1.1) containing a His-tag was heterologously expressed in *Escherichia coli* and purified as reported earlier (Maier et al., 2018; Greifenstein et al., 2022). Aliquots were stored at −80°C until use.

### 2.3 Bioink preparation

Aqueous solutions of agar and low-melt agarose (3–6% (w/w)) were prepared by adding appropriate amounts of agar or agarose powder to PBS buffer and heating the mixture in a microwave oven (WP800L20-5, Hanseatic) several times to boiling point, until a homogeneous solution was obtained. The solution was transferred into a 25 ml SpeedMixer^®^ cup (Hauschild GmbH & Co. KG) and the temperature was adapted to 65°C in a water bath. If required, the appropriate amount of AaEst2 stock solution was added to obtain the desired enzyme concentration of 100 nM. Due to its limited availability, no enzyme was added, if the bioink was intended for rheological analysis, mechanical testing or diffusibility measurements. It was assumed that the low concentrations of enzyme used (100 nM) did not significantly influence the evaluated material properties. The bioink was mixed and degassed in a dual asymmetric centrifuge (DAC) SpeedMixer^®^ DAC 150.1 FVZ-K (Hauschild GmbH & Co. KG) at 3,500 rpm for 90 s. Before the mixing step, the cup holder of the SpeedMixer^®^ was pre-heated to 65°C to avoid gelation during the mixing step. Bioinks intended for printing were transferred into pre-heated 10 ml printing cartridges, sealed with an outlet cap and a piston (all purchased from Nordson EFD) and used directly. Bioinks intended for enzyme-free analytics were filled into 10 ml syringes and stored at 65°C until use.

### 2.4 Rheology

The flow properties and gelation behavior of the bioinks were analyzed using an MCR 301 rheometer (Anton Paar GmbH). To prepare a measurement, liquid bioink was applied to the pre-heated rheometer plate at 60°C. The top plate was moved to the measurement position and the sample was trimmed. To avoid air exposure and prevent the sample from drying out during the measurement, paraffin oil (Fluka Analytical) was applied to the measurement gap. The temperature was set to the required start temperature of the measurement and the sample was left to equilibrate for at least 5 min.

#### 2.4.1 Gelation behavior

Temperature hysteresis curves were recorded to investigate the melting and gelling behavior of the bioinks. The measurements were performed with profiled parallel plates with a diameter of 25 mm and a gap width of 500 µm. The measurement was started at 70°C. Gelation was induced by steadily decreasing the temperature to 15°C at a rate of 1°C  min^−1^. To remelt the sample, the temperature was increased at the same rate to 80°C for low-melt agarose and to 100°C for agar. During the whole process, the storage modulus *G*′ and the loss modulus *G*″ were recorded applying an oscillatory measurement with a shear strain (deformation) amplitude *γ*_*A*_ = 3% and an angular frequency *ω* = 20 s^−1^. Each data point was averaged over a period of 30 s. All measurements were carried out as triplicates (*n* = 3).

To determine the melting temperature (*T*_*melt*_) and gelling temperature (*T*_*gel*_), an inflection point method derived from Bonino et al. (2011) was applied. The descending and ascending part of the *G*′ measurement were separately fitted with a sigmoidal curve and the inflection point of the curve was defined as *T*_*melt*_ and *T*_*gel*_, respectively.

#### 2.4.2 Flow behavior

Flow curves of the bioinks were recorded at different temperatures to gather information about the behavior of the inks during extrusion. A cone-plate setup with a diameter of 60 mm was employed. The viscosity was determined using shear rate-controlled rotational measurements. All inks were analyzed in triplicates (*n* = 3) at a comparison temperature *T*_*c*_ = 70°C and the specific nozzle temperature *T*_*nozzle*_ of the respective ink, as shown in Table 1. To observe the influence of temperature on flow behavior in the range relevant during the printing process, the same measurement was performed in the range from 40°C to 25°C for low-melt agarose and from 50°C to 35°C for agar, both in 1°C steps with one measurement per temperature (*n* = 1). The measurements were performed with a variable measuring time per data point, ranging from 10 s at a shear rate of 0.1 s^−1^ to 1 s at 1000 s^−1^.

**TABLE 1:** Printing parameters employed in the fabrication of activity assay cylinders and exemplary prints made from inks based on low-melt agarose and agar.

Polymer type	Polymerconcentration(% (w/w))	Printingspeed(mm/s)	Extrusionpressure^a^(kPa)	Cartridgetemperature^b^(°C)	Nozzletemperature(°C)
Agar	3	5	30	52	47
	4.5	7	120	60	55
	6	7	300	65	57
Agarose	3	5	40	38	30
	4.5	7	160	39	32
	6	7	380	40	35

^a^ The extrusion pressure was continuously adapted to produce cylinders of the specified target weight.

^b^ The actual (measured) temperature inside the cartridge was roughly 3°C lower than the set value.

### 2.5 3D bioprinting

#### 2.5.1 Customized experimental setup

**FIGURE 2:**
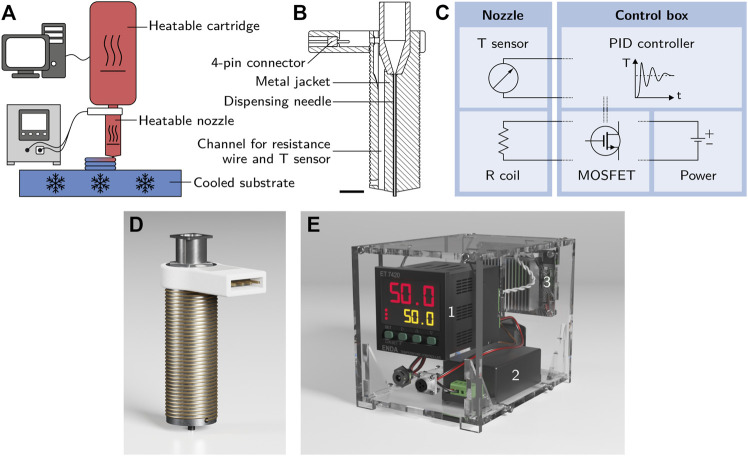
**(A)** Scheme of the printing setup with a heatable cartridge, a customized heatable nozzle and a cooled substrate. **(B)** Cross-section of the metal jacket of the heatable nozzle before the assembly of resistance wire and temperature sensor (the scale bar represents 5 mm). **(C)** Simplified circuit layout of the setup showing the interaction of the nozzle containing a temperature sensor and a resistance coil with the control box containing a PID controller, a power supply and a MOSFET control board. **(D)** 3D illustration of the heatable nozzle used for the printing process. The assembled nozzle consists of a 3D-printed steel body containing a commercially available 2 inch steel dispensing tip with an inner diameter of 0.35 mm and a Pt 100 element as a temperature sensor. A resistance wire is coiled around the body of the nozzle for heating. **(E)** Control box containing a PID controller 1), a 12 V power supply 2) and a MOSFET control board 3) to regulate the temperature of the heatable nozzle.

All bioinks were printed using a BioScaffolder 3.1 (GeSiM—Gesellschaft für Silizium-Mikrosysteme mbH) with a specifically adapted setup including a custom-made heatable nozzle and a water-cooled microplate carrier. A scheme of the setup is shown in Figure 2A. The custom-made heatable nozzle is depicted in Figure 2B as a cross-section and in Figure 2D as a 3D illustration. A commercially available 2 inch steel dispensing tip (Vieweg GmbH) with an inner diameter of 0.35 mm and a length of 25 mm was the basis of the heatable nozzle. It was placed inside a 3D-printed metal jacket that contained a Pt 100 element (L220, Heraeus Deutschland GmbH & Co. KG) to measure the nozzle temperature. Heating was achieved with a resistance wire coiled around the metal jacket. The components of the heatable nozzle were joined with thermo-conductive epoxy resin (Omegabond 200, Omega Engineering GmbH). The temperature sensor and resistance wire were coupled with a control box containing a 12 V, 60 W power supply (IRM-60-12ST, Mean Well Enterprises Co., Ltd.), a MOSFET board and a PID controller (ET 7420, ENDA GmbH & Co. KG). The output from the temperature sensor was processed by the PID controller to operate the MOSFET board which regulated the current flow in the resistance wire. This allowed the metal body of the nozzle to be kept at the set temperature. A simplified circuit layout of the setup is shown in Figure 2C, a 3D illustration of the control box in Figure 2E. Sand-blasted glass plates were used as the printing substrate. To accelerate the gelation process, the plates were cooled to 5°C by a water-cooled microplate carrier connected to an F12-MP cooling aggregate (Julabo GmbH).

#### 2.5.2 Printing process

Immediately after the preparation process, the temperature of the bioink cartridges was adjusted to the final printing temperature by incubation in the heating jacket of the BioScaffolder for 20 min at the temperature appropriate for the corresponding bioink. The pre-heated nozzle was mounted onto the cartridge, calibrated for height and purged with bioink. Prints were always carried out at a fixed layer height of 300 μm, all other printing parameters were individually adjusted to the corresponding inks as shown in Table 1.

Hollow, enzyme-loaded hydrogel cylinders were printed to perform activity assays with different hydrogel compositions and substrate concentrations in multiwell plates (see Section 2.8.1). For mechanical tests (Section 2.6), identical cylinders were printed without added enzyme. The cylinder diameter of 10 mm, the total height of 3 mm and the layer height of 300 µm were kept identical for all prints, independent of the used ink. In order to produce comparable cylinders, every printed cylinder was weighed and the extrusion pressure was continuously adapted in order to yield a target weight of 100 mg per cylinder. Cylinders not meeting the specified target weight within a ±3% tolerance window were discarded. Cylinders matching the defined specifications were stored in multiwell plates (CellStar^®^ 48 well suspension culture plate, Greiner Bio-One GmbH) sealed with self-adhesive plastic foil (polypropylene, for PCR plates, Brand GmbH & Co. KG).

### 2.6 Assessment of cylinder height and mechanical properties of hydrogels

A ZwickiLine Z0.5TN universal testing machine (ZwickRoell GmbH & Co. KG) equipped with a 100 N load cell (Xforce HP) and stainless steel compression plates with a diameter of 30 mm was used to determine the height and maximum tolerable force of printed hydrogel cylinders with uniaxial compression tests. These measurements can provide valuable information about the mechanical stability of the hydrogels which determines the degree of stress they can tolerate during handling. All measurements were controlled employing the software testXpert III, V.1.4 (ZwickRoell GmbH & Co. KG). For the measurement, a sample was placed on the bottom plate of the device. Every measurement was started at a gap width of 3.5 mm and the top plate with the load cell was moved down at a speed of 2 mm/min. The measurement gap at a load of 0.01 N was defined as the cylinder height. The maximum achievable compression force before a rupture of the sample occured was defined as *F*_max_. As a comparison with non-printed samples, the maximum force measurement was also performed with solid hydrogel cylinders punched out of a 3 mm thick layer of cast hydrogel. Twelve samples were measured for each data point (*n* = 12).

### 2.7 Diffusion properties

The diffusion coefficient of 5(6)-carboxyfluorescein in different hydrogels was estimated employing a microfluidics-based UV imaging method reported previously (Wenger and Hubbuch, 2022). A solution of 1 mg/ml 5(6)-carboxyfluorescein (Acros Organics, part of Fisher Scientific Co. LLC) in 6.25% (v/v) dimethyl sulfoxide (DMSO, purchased from Fisher Scientific Co. LLC) in PBS was prepared by dissolving the appropriate amount of 5(6)-carboxyfluorescein in pure DMSO and then diluting it with the appropriate amount of 3.125% DMSO in PBS. The microfluidic chip was pre-heated to 70°C in a drying oven (T 6120, Heraeus Instruments GmbH & Co. KG), filled with liquid bioink and left at room temperature for 10 min to allow gelation. The 1 mg/ml 5(6)-carboxyfluorescein solution was filled into the chip through one of the other inlets to create an interface between solution and hydrogel. The diffusion of the 5(6)-carboxyfluorescein through the hydrogel was monitored using an ActiPix™ D100 imaging system (Paraytec Ltd.). The resulting raw data were transformed to absorbance data and exported as wmv files using the ActiPix™ software (version 1.5). Matlab R2020a (The MathWorks^®^, Inc.) was used to import the files, detect the channel area and rotate the frames in order to achieve a horizontal alignment of the channel. Using a calibration curve, the image data at 120 min were converted to 5(6)-carboxyfluorescein concentration data. To obtain a value for the diffusion coefficient *D*, the concentration profiles along the microfluidic channel were fitted with an analytical solution of Fick’s second law (Wenger and Hubbuch, 2022):

$$C\left(x,t\right)=C_{0}\left(\frac{1}{2}-\frac{1}{2}\mathrm{erf}\left(\frac{x-x_{0}}{2\sqrt{Dt}}\right)\right)$$ (1)

with the position along the microfluidic channel *x*, the time *t*, the initial concentration of 5(6)-carboxyfluorescein in the fluid phase *C*_0_, the position of the boundary layer *x*_0_ and the diffusion coefficient *D*. All measurements were performed as triplicates (*n* = 3).

### 2.8 Activity assays

#### 2.8.1 Enzyme immobilized in hydrogels

Printed hydrogel cylinders were assessed for their enzymatic activity in a 48-well microplate format using a Tecan Freedom Evo pipetting platform (Tecan Group AG). Each well of a 48-well microplate (for suspension culture, Greiner Bio-One GmbH) contained one printed hydrogel cylinder loaded with 100 nM esterase 2 (AaEst2). For the measurement, 320 µl of substrate solution (10–150 µM 5(6)-carboxyfluorescein dihexylester in PBS, pH 7.4) were added to each cylinder using the Tecan Freedom Evo pipetting robot. To avoid evaporation effects, 300 µl of light mineral oil (Sigma-Aldrich, part of Merck KGaA) were added manually using a multichannel pipette. Calibration samples of 5(6)-carboxyfluorescein (0–150 µM) were prepared in the same way on the same plate to allow the transformation of fluorescence measurements to concentration values. The fluorescence signal (*λ*_*excitation*_ = 485 nm, *λ*_*emission*_ = 528 nm) was recorded for 2 h in a Tecan infinite M200 pro spectrophotometer. Using the obtained calibration curves, the resulting fluorescence measurements were converted to concentration values and the curves of 5(6)-carboxyfluorescein concentration over time were fitted with sigmoidal fits. To determine the maximum activity of a sample, the maximum slope of the fits was determined. The activity assays were performed in triplicates (*n* = 3) at 25°C with different hydrogel compositions (3%, 4.5% and 6% (w/w) low-melt agarose and agar).

#### 2.8.2 Freely dissolved enzyme leached from hydrogels

During the activity assays with enzyme-loaded hydrogel cylinders, six cylinders were incubated with only PBS buffer (substrate-free). At the end of the measurement after 2 h, the supernatants were sampled and stored for later analysis of their catalytic activity as an indicator of leaching. The activity assays were conducted in the same way as with the printed hydrogel cylinders, i.e., employing a Tecan Freedom pipetting platform and 48-well microplates. 320 µl of 50 µM substrate solution were added to 100 µl of supernatant to induce the catalytic reaction. 300 µl of light mineral oil (Sigma-Aldrich) were added manually to avoid evaporation effects. Calibration samples of 5(6)-carboxyfluorescein (0–150 µM) were prepared in the same way on the same plate to allow the transformation of fluorescence measurements to concentration values. The fluorescence signal (*λ*_*excitation*_ = 485 nm, *λ*_*emission*_ = 528 nm) was recorded for 20 min at 25°C. The resulting data were converted to product concentrations using calibration curves. The volumetric activity was determined from the initial slope (over 5 min) of the product-over-time curve. All measurements were performed as triplicates (*n* = 3).

### 2.9 Statistical analysis

The statistical significance of data was tested employing one-way analysis of variance (ANOVA) and the Tukey method for multiple comparisons. Differences between data points were considered statistically significant when *p* < 0.05. Normality was assessed for large data sets (*n* = 12) applying the Anderson-Darling test. For small data sets (*n* = 3), normality was assumed.

## 3 Results and discussion

### 3.1 Rheology and printability

The print fidelity of extrusion-based 3D printing mainly depends on the extent of ink spreading after extrusion which is primarily influenced by two parameters: the rheological properties of the liquid bioink, namely the viscosity or the presence of a yield point, and the time delay, until the ink is solidified after extrusion (Jungst et al., 2016). For thermosensitive materials like agarose or agar-based inks, both properties are substantially influenced by temperature. Excessively high printing temperatures may reduce the viscosity and prolong the period of ink spreading, while too low temperatures may allow premature gelation to occur within the nozzle resulting in the extrusion of distorted or currugated filaments (Mouser et al., 2016; Gu et al., 2018), nozzle clogging (Ozbolat and Hospodiuk, 2016; Gu et al., 2018) and reduced inter-layer adhesion (Ozbolat et al., 2014; MacCallum et al., 2020). Hence, the thermal regulation of both cartridge and nozzle is paramount in order to achieve the desired ink properties upon extrusion. For this purpose, a customized printer setup with a heatable nozzle and a heatable cartridge jacket was employed. Rheological methods were used to study the thermo-dependent behavior of agarose and agar inks. In particular, the gelling and melting behavior and the influence of temperature on flow properties were analyzed.

#### 3.1.1 Gelling and melting behavior

**FIGURE 3:**
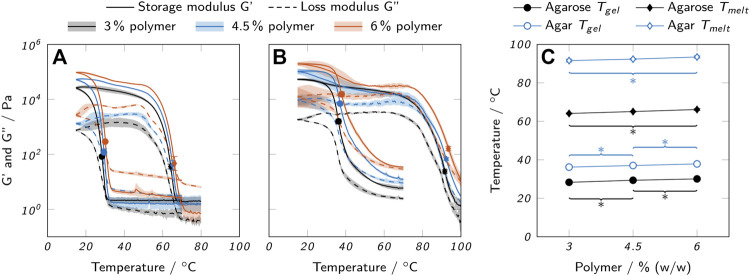
Thermal properties of low-melt agarose and agar hydrogels. Loss and storage modulus of **(A)** low-melt agarose and **(B)** agar hydrogels are shown over temperature. **(C)** Melting and gelling points of low-melt agarose and agar, as derived from the oscillatory measurements. All data are shown as mean values with the standard deviation as shaded areas or error bars (*n* = 3). For clarity, only significant differences to the nearest significantly different data points are highlighted by asterisks (*p* < 0.05).

Gelling and melting temperatures of the bioinks were determined using oscillatory measurements in combination with temperature sweeps, as shown in Figure 3. Liquid bioink samples were applied to the pre-heated rheometer plate at 60°C and solidified by cooling below the gel point *T*_*gel*_, indicated by a sudden increase in storage modulus *G*′ and loss modulus *G*″. The samples were reliquefied by increasing the temperature above the melting point *T*_*melt*_, accompanied by a decrease in *G*′ and *G*″. The gel point is commonly determined by calculating the cross-over of *G*′ and *G*″ (Billiet et al., 2014; Wüst et al., 2014; Mao et al., 2016). In the given case, some samples displayed *G*′ > *G*″ over the whole analyzed range and there was no cross-over despite the obviously liquid nature of the samples at 70°C. Mao *et al.* found that incorrectly high values of *G*′ may be caused by oxidation effects on the surface of the metallic measuring plates or by a partial invasion of the measurement gap by oil that is used to protect the sample from evaporation (Mao et al., 2016). This invasion may be further promoted by thermal expansion and contraction effects during the temperature sweep. Hence, an alternative approach reported by Bonino et al. (2011) was applied to estimate the gelling and melting temperatures from the inflection points of the *G*′ curves as indicated in Figures 3A,B. The final estimations of *T*_*melt*_ and *T*_*gel*_ are shown in Figure 3C.

Agarose bioinks exhibited a very sharp increase in *G*′ upon gelation, while the change occured more gradually with agar bioinks. Both agarose and agar inks exhibited a strong thermal hysteresis, i.e. a large difference between *T*_*gel*_ and *T*_*melt*_. While the polymer concentration was found to have a relatively low impact on *T*_*gel*_ and *T*_*melt*_ with a maximum difference of 2°C between 3% and 6% polymer, there was a strong influence of the material system. In general, agar bioinks gelled and melted at higher temperatures (*T*_*gel, mean*_ = (37.0 ± 0.8)°C and *T*_*melt, mean*_ = (92.5 ± 0.9)°C) than agarose bioinks (*T*_*gel, mean*_ = (29.3 ± 0.9)°C and *T*_*melt, mean*_ = (65.1 ± 1.0)°C). The determined values for agarose were in good accordance with manufacturer’s data (*T*_*gel*_ ≤ 28°C and *T*_*melt*_ ≤ 65.5°C for a 1.5% gel).

The determined gelling and melting temperatures were used as a basis to select suitable temperatures for printing. Commonly, thermosensitive hydrogels are printed in a partially cross-linked state at a temperature close to the cross-over point of *G*′ and *G*″ in order to reduce spreading after extrusion but still allow for a smooth dispensing process (Laronda et al., 2017). We found that operating at temperatures too close to *T*_*gel*_ resulted in a time-dependent increase in viscosity and finally nozzle clogging due to slow gelation within the cartridge, especially with agar inks. Thus, the final adjustments of nozzle and cartridge temperatures were done in an iterative process. Higher temperatures were chosen for the cartridge than for the nozzle to avoid time-dependent gelation and to ensure constant extrusion conditions. The tendency of agar to gel prematurely increased strongly with growing concentration resulting in a high temperature difference between different agar concentrations, while different low-melt agarose inks were printed at relatively similar temperatures (see Table 1). Also, it was found that the actual ink temperature within the cartridge was roughly 3°C lower than the set value which was considered, as well. The selected temperatures are listed in Table 1.

The recorded temperature sweeps also allow an assessment of the possible application temperatures for biocatalytic reactions with enzymes immobilized in the printed hydrogels. The chosen temperature should allow the enzyme to work as efficiently as possible while not impairing the mechanical integrity of the hydrogel. AaEst2 shows an activity maximum at a temperature of approximately 70°C (Manco et al., 1998). At a temperature of 25°C, as employed for all experiments in this study, the activity is lower by a factor of approximately 4.5 (Manco et al., 1998). To avoid a weakening of the hydrogel, the reaction should be performed at a temperature below the onset of the melting process which is around 50°C for the agarose hydrogels and around 70°C for the agar hydrogels. Hence, the use of agar hydrogels would allow the AaEst2 to operate at its optimum temperature while reactors made from agarose hydrogels can only be operated at suboptimal temperatures. It should be noted that a modified agarose with low melting point was used in this study and higher operating temperatures could be realized with a different type of agarose.

#### 3.1.2 Flow properties

**FIGURE 4:**
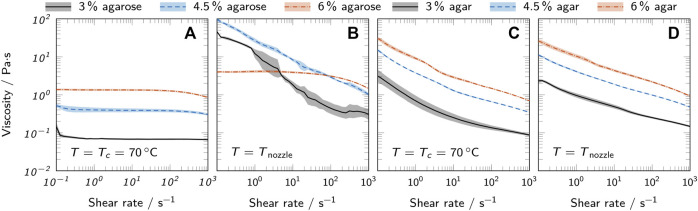
Viscosity curves of all prepared bioinks at 70°C and the respective nozzle temperature *T*_*nozzle*_ used for printing. Low-melt agarose bioinks at **(A)** 70°C and **(B)** *T*_*nozzle*_ are compared to agar bioinks at **(C)** 70°C and **(D)** *T*_*nozzle*_. All curves show mean values and the standard deviation as shaded areas (*n* = 3).

**FIGURE 5:**
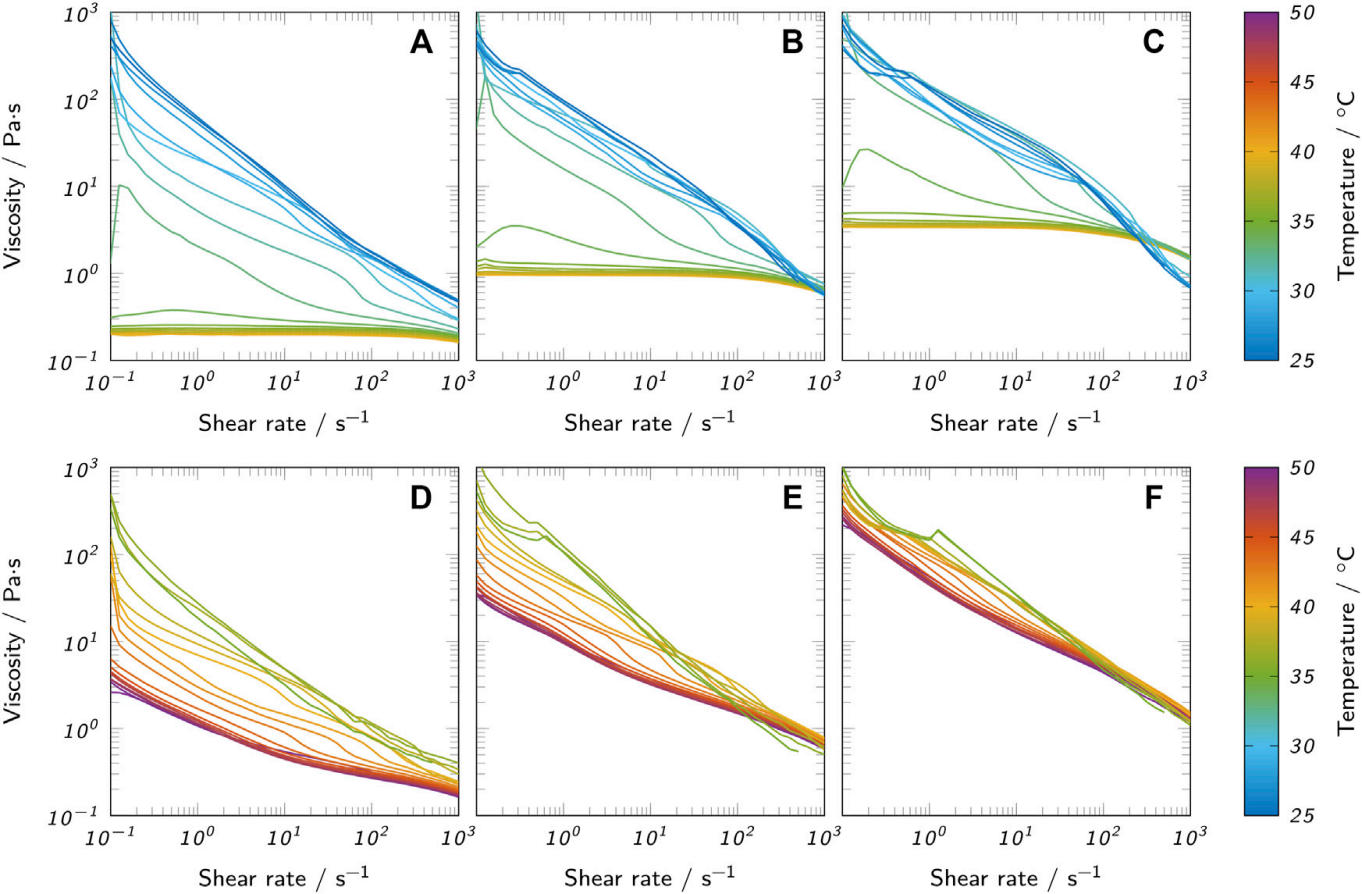
Flow curves of all prepared bioinks at different temperatures. **(A)** 3% low-melt agarose, **(B)** 4.5% low-melt agarose, **(C)** 6% low-melt agarose, **(D)** 3% agar, **(E)** 4.5% agar and **(F)** 6% agar.

The flow properties of liquid agarose and agar inks were analyzed using rotational tests. Figure 4 shows the viscosity over shear rate for both a comparison temperature *T*_*c*_ = 70°C and the nozzle temperature *T*_*nozzle*_ which was specific for each bioink (see Table 1). The temperature range relevant for printing was covered in more detail by additional measurements, as represented in Figure 5. Here, the flow curves recorded at different temperatures are shown in the range of 40–25°C for agarose inks and 50–35°C for agar inks. In general, all inks showed a strong correlation between polymer concentration and viscosity. At *T*_*c*_, agarose inks exhibited ideally viscous (Newtonian) behavior in the analyzed range, i.e. a constant viscosity, independent of the applied shear rate. Lowering the temperature to *T*_*nozzle*_ drastically changed the behavior of agarose inks with 3% and 4.5% polymer towards the shear-thinning behavior of a pseudo-plastic fluid. The 6% agarose ink still showed Newtonian behavior at shear rates below 100 s^−1^ with a viscosity plateau shifted up by a factor of 3 compared to *T*_*c*_. The seemingly inconsistent behavior at *T*_*nozzle*_ can be attributed to the different nozzle temperatures used for different agarose concentrations. When reducing the temperature further, the same shear-thinning behavior was observed for 6% agarose as for the other concentrations (see Figures 5A–C). The found shear-thinning behavior of most inks at *T*_*nozzle*_ is a favorable property for extrusion-based bioprinting, as it contributes to high-fidelity printing (Chimene et al., 2016).

The measurements with variable temperature (Figure 5) confirm the observation of the oscillatory measurements that the change in material properties is more abrupt in agarose inks than in agar inks when approaching *T*_*gel*_. This observed change of rheological behavior with the reduction of temperature can be explained by the onset of gelation. At high temperatures, the agarose polysaccharide chains behave as random coils. During cooling, the chains start forming double-stranded helices stabilized by intramolecular hydrogen bonds. At further reduced temperatures, the helices start to aggregate due to intermolecular hydrogen bonds, forming microcrystalline junction zones (Tako and Nakamura, 1988; te Nijenhuis, 1997; Fernández et al., 2007). The gelation continues with reduced temperature and leads to a sharp increase in viscosity when approaching the gel point (Fernández et al., 2007) causing the observed shear-thinning behavior. Agar inks already showed shear-thinning behavior at 70°C and only marginally changed their rheological properties upon temperature reduction to *T*_*nozzle*_ which can be attributed to the larger difference between *T*_*nozzle*_ and *T*_*gel*_ for agar inks.

#### 3.1.3 Printability

**FIGURE 6:**
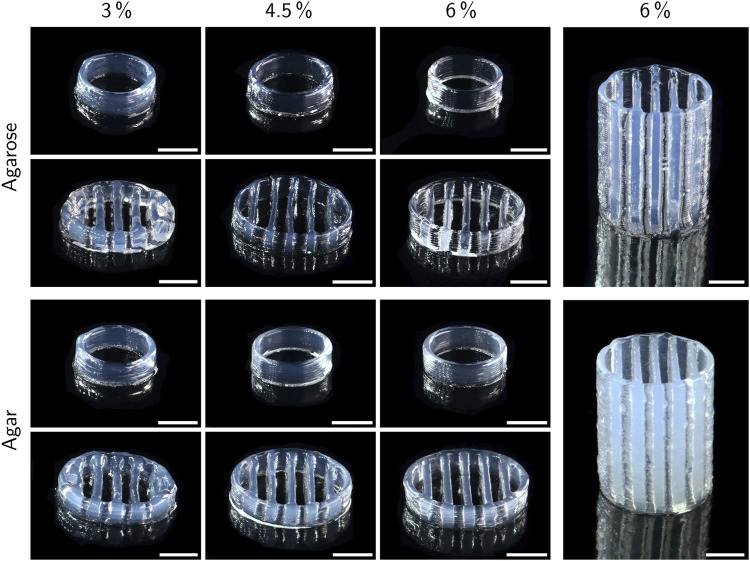
Exemplary prints of low-melt agarose and agar hydrogels with a target height of 3 mm (left side) and 20 mm (right side). All prints were carried out at a layer height of 300 µm. Scale bars represent 5 mm.

The printability of different bioinks was compared by printing two standardized test objects, namely a hollow cylinder and a lattice structure. All prints were carried out at a layer height of 300 µm with a target height of 3 mm. Additionally, the lattice structure was printed with a target height of 20 mm only using inks with 6% polymer. Figure 6 shows the resulting prints. For both agarose and agar inks, the printability was drastically enhanced at higher polymer concentrations, especially for the lattice structure. While at 3% polymer, the circular outline merged almost entirely with the outermost transverse strands, there was a clear separation between outline and inner strands in prints with 4.5 and 6% polymer and the extruded strands were thinner in general. The increased spreading of inks with 3% polymer did not allow printing structures notably higher than 3 mm with the employed settings. Lowering the printing speed or the nozzle and cartridge temperature may reduce the spreading effect and allow the printing of higher objects. More efficient cooling, e. g. employing a fan, might also contribute, but comes with the drawback of accelerating the drying of the gel. Grid structures with more layers were printable only with inks containing 4.5 and 6% polymer. Figure 6 shows grids with a height of 20 mm made from inks with 6% agarose and agar. In general, the resolution of the printed objects was relatively low with a strand thickness of roughly 600–1,000 µm which could be optimized by adding suitable additives like nanosilicates to enhance the viscosity of the inks. At large, the presented results are in accordance with the rheological analysis, as lower viscosities caused reduced printing quality due to increased ink spreading. Despite a lower measured viscosity at *T*_*nozzle*_ at low shear rates, the 6% agarose ink showed superior printability compared to 3% agarose. This behavior contradicts the rheological observations indicating that the actual extrusion temperature deviated from *T*_*nozzle*_. The inks were stored in the cartridge at a slightly higher temperature (*T*_*cartridge*_) to prevent time-dependent gelation effects. The residence time of ink within the nozzle was calculated to be between 1.1 s and 1.5 s (see supplementary material) which may have been too short to cool the ink to *T*_*nozzle*_ resulting in a slightly higher extrusion temperature. Small temperature differences close to *T*_*gel*_ can cause strong variations in viscosity (Fernández et al., 2007) and hence influence the printability significantly. Faster gelation kinetics of the high-polymer inks may play an additional role.

**FIGURE 7:**
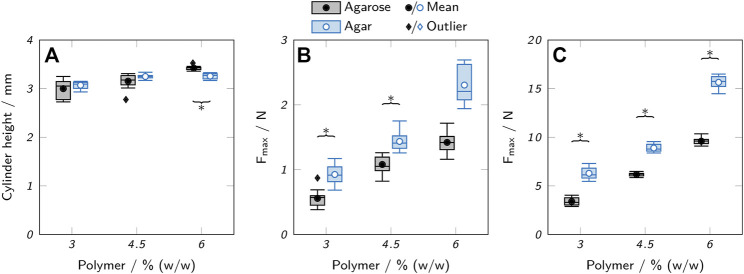
Mechanical testing of low-melt agarose and agar hydrogels. **(A)** Height of printed hydrogel cylinders. **(B,C)** Maximum applicable compression force *F*_max_ before rupture of **(B)** printed hollow hydrogel cylinders and **(C)** cast hydrogel samples. The box plots represent the median and the upper and lower quartile. The whiskers represent the most extreme value still within a 1.5-fold interquartile range (IQR) from the upper and lower quartile. All data points outside the 1.5-fold IQR are depicted as outliers. Each box represents twelve samples (*n* = 12). Significant differences between agarose and agar are highlighted by asterisks (*p* < 0.05).

Compared to the lattice structures, the basic hollow cylinders only showed minor differences between different polymer concentrations. At 4.5 and 6% polymer, the single layers were more visible in form of a ribbed surface, indicating a higher surface area available for mass exchange compared to inks with 3% polymer, where the layers were more smoothly merged due to increased ink spreading before gelation. Excessive ink spreading can also cause a deviation of the actual object height from the target height defined by the executed gcode. Height measurements with a universal testing machine indeed revealed a positive correlation between polymer concentration and the height of printed cylinders, as shown in Figure 7A. The maximum difference in height of 14% was found between cylinders made from 3 to 6% agarose. Assuming a perfect hollow cylinder, this corresponds to a difference of 8.7% in surface area available for mass exchange (see supplementary material).

The presented photographs only allow a qualitative assessment of printability. For further studies with adapted ink compositions it would be desirable to apply more precise analysis methods that allow an objective assessment based on quantitative data.

### 3.2 Material properties of agarose and agar hydrogels

#### 3.2.1 Mechanical strength

High mechanical strength is not a primary requirement for hydrogels employed in a biocatalytic reactor unless high operating pressures or shear forces are involved. However, weak hydrogels can massively impede the handling of printed objects and complicate processing steps like the reactor assembly or the removal of the printed hydrogel from the printing substrate. As a measure of mechanical strength, the maximum compression force *F*_max_ before rupture of the gels was determined using a universal testing machine. Printed hollow cylinders, as used for the printability and activity studies, were compared to solid cylinders punched out of a layer of cast hydrogel, as shown in Figures 7B,C. Due to the different geometries of the cast and printed samples (solid vs. hollow cylinders), only trends can be compared, not the absolute values of the measurements.

If extracted from the same source and used at the same concentration, agarose forms stronger gels than agar (Zhang et al., 2019) due to the lack of non-gelling components like agaropectin (Selby and Whistler, 1993). Here, agar hydrogels showed a higher tolerable compression force than the respective agarose gels by a factor of 1.3–1.9. This is probably due to the use of an agarose with low melting and gelling point, prepared by introduction of hydroxyethyl groups into the agarose skeleton which is associated with a reduction in gel strength (Zhang et al., 2018). The polymer concentration was positively correlated with *F*_max_ with a 2.5-fold increase between 3% and 6% (w/w) polymer for agar hydrogels, independent of the preparation method. An increased polymer concentration allows the formation of more junction zones between agarose chains and hence higher stability (Arnott et al., 1974). For agarose hydrogels, *F*_max_ increased by a factor of 2.8 for cast samples, but only by a factor of 2.5 for printed samples. This implies that differences in geometry play an additional role in the stability of the printed cylinders. Reduced contact areas in samples printed with high-viscosity inks may weaken the integrity of the printed object, while the increased layer merging of low-viscosity inks may be beneficial for the stability. In general, the measurements of the printed objects showed higher standard deviations than the samples punched-out from cast material. This indicates a lower reproducibility and larger deviations between theoretically identical printed samples which may be influenced by geometric irregularities due to an unsteady ink flow or material inhomogeneities caused by changing cooling patterns.

It is important to note that the mechanical testing was performed at room temperature and that employing the hydrogels at elevated temperatures to optimize the enzymatic activity may drastically reduce the mechanical stability, as discussed in Section 3.1.1. Another aspect of mechanical stability of printed objects is the adherence between layers which was not analyzed quantitatively. However, it was made sure that the printing temperatures were chosen in a way that guaranteed the ink to come in contact with the previous layer in a non-gelled state. This allowed the handling of the printed objects like the transfer from the printing substrate into microplates without any delamination. Also, the performed compression tests always caused a vertical rupture of the printed cylinders indicating that the interlayer bonding was not a particular weak spot.

#### 3.2.2 Diffusion characteristics

**FIGURE 8:**
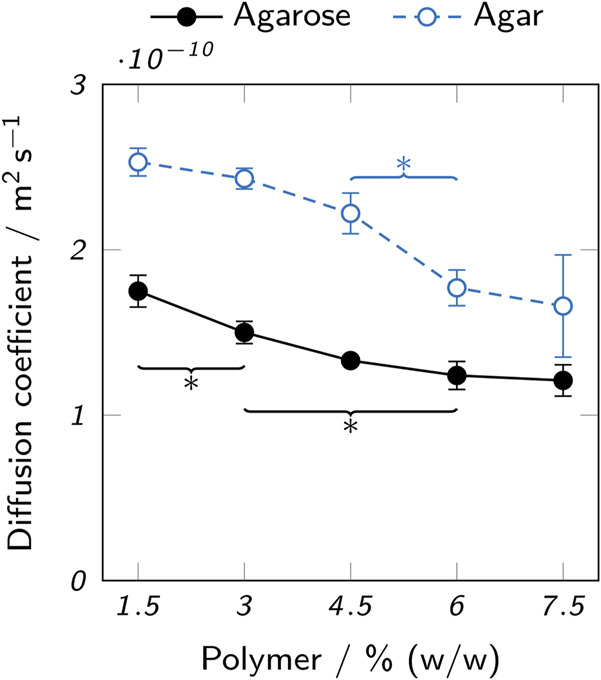
Diffusion coefficients of 5(6)-carboxyfluorescein in low-melt agarose and agar hydrogels. The data points represent mean values ± standard deviation (*n* = 3). For clarity, only significant differences to the nearest significantly different data points are highlighted by asterisks (*p* < 0.05).

The immobilization of enzymes by physical entrapment in hydrogels leads to reduced catalytic efficiency due to mass transfer limitations caused by the hydrogel (Schmieg et al., 2018; Schmieg et al., 2020; Wenger et al., 2020). To reduce this effect, a high diffusibility of the hydrogel itself and short path lengths due to appropriate geometries of the printed object are desirable. The diffusion coefficient of 5(6)-carboxyfluorescein in agarose and agar hydrogels was determined to assess the effect of different polymer types and concentrations on mass transfer limitations. 5(6)-carboxyfluorescein is the product of the cleavage of 5(6)-carboxyfluorescein dihexylester by AaEst2 and was chosen as the analyte, as it is readily available and can be detected using UV-Vis spectroscopy. Figure 8 shows the results of the measurements over a range of 1.5–7.5% (w/w) polymer. A decrease of the diffusion coefficient with increasing polymer concentration could be observed for both hydrogel types. Over the whole analyzed range, the diffusion coefficient in agar hydrogels was higher than in agarose hydrogels by a factor of 1.4–1.7. For both, the diffusion coefficient dropped by roughly 30% between 1.5% and 7.5% (w/w) polymer.

The observed higher diffusion coefficients in agar hydrogels can be attributed to the expected smaller pore size of low-melt agarose hydrogels. Both the lack of non-gelling components like agaropectin (Selby and Whistler, 1993) in agarose and the chemically modified polymer chains of the low-melt agarose contribute to this effect (Cook, 1982). The pore sizes of agarose hydrogels reported in literature are typically by orders of magnitude larger than small molecules like 5(6)-carboxyfluorescein ((289 ± 66)nm in 3% agarose, (201 ± 36)nm in 5% agarose (Maaloum et al., 1998)). However, increased path lengths due to steric obstruction by the polymer chains and hydrodynamic drag can reduce the diffusion coefficient (Amsden, 1998). Furthermore, not only the type of agarose, but also properties of the solvent like ionic strength and process parameters like cooling speed can strongly influence the molecular structure and hence diffusivity of the resulting hydrogel (Maaloum et al., 1998). Specifically, low-melt hydroxyethyl agarose is known to form smaller pores than the unmodified starting product (Cook, 1982) and rapid cooling creates a more uniform agarose microstructure with thinner fibers and smaller average pore diameters than slow cooling (Kusukawa et al., 1999). In practice, the cooling process is hard to control and can even lead to a heterogeneous pore distribution within a single sample, as some parts will cool and gel more quickly due to contact with a cold surface, while other parts are cooled slower by the contact with air or because the core will cool slower than the surface. This leads to a limited comparability between the samples analyzed here and printed objects.

### 3.3 Enzyme immobilization within printed agar and agarose hydrogels

The achievable catalytic activity and the amount of enzyme leaching from the material are relevant criteria to assess the suitability of the studied hydrogels for the application in printed biocatalytic reactors. Both these properties were examined in microplate-based batch experiments with hollow printed hydrogel cylinders containing 100 nM AaEst2. In comparison to cast samples, printed hydrogel cylinders offer a more realistic representation of a printed reactor due to the identical production process and hence similar material properties. During the production process, the enzyme is exposed to a certain regime of temperature changes which cannot be replicated by using cast samples and which is especially relevant when using enzymes that are susceptible to thermal inactivation. Material properties determined by the production process include geometric differences caused by varying degrees of layer merging or differences in the polymer structure of the hydrogels as a result of printing temperature and cooling rate during gelation (Maaloum et al., 1998; Mohammed et al., 1998; Kusukawa et al., 1999). Thus, the results presented here are to be interpreted as a function of not only polymer type and concentration but also various other process- and material-related parameters.

#### 3.3.1 Leaching

**FIGURE 9:**
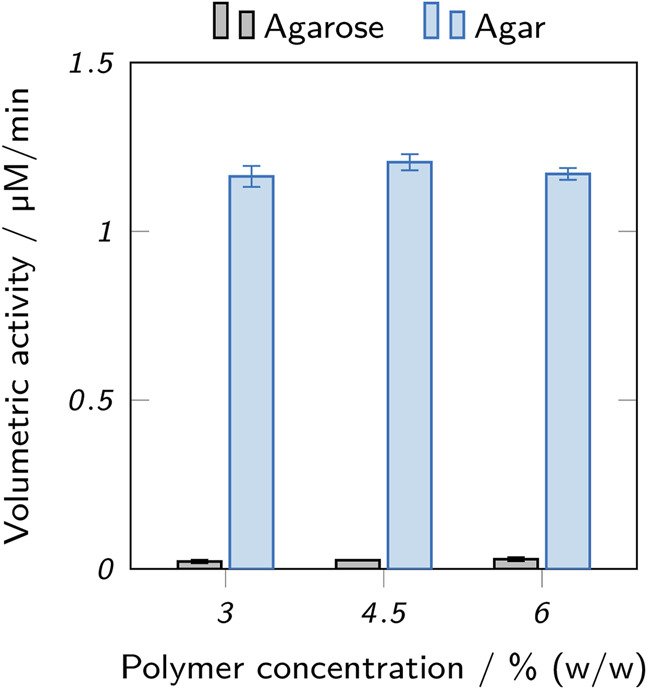
Activity assay of supernatants incubated with enzyme-laden hydrogel cylinders made from inks based on low-melt agarose and agar. Different amounts of leaching can be observed for different hydrogels. The data points represent mean values ± standard deviation (*n* = 3). Significant differences between polymer concentrations are marked by asterisks (*p* < 0.05).

Enzyme-laden hydrogel cylinders were immersed in buffer for 120 min and samples of the supernatants were analyzed for leached enzyme using activity assays. Figure 9 shows the acquired results. Only a low amount of enzymatic activity was observed in the supernatants of agarose hydrogels (below 0.03 μM/min), but values between 1.16 μM/min and 1.20 μM/min were determined for the supernatants of agar hydrogels. For both hydrogel types, no significant differences between different polymer concentrations were found. The substantial enzyme leaching observed for agar hydrogels indicates that no sufficient enzyme retention was achieved. In perfusable reactors, the degree of leaching would be even higher than in the static experiment performed here. This demonstrates the poor suitability of agar hydrogels in this context.

The immobilization of enzymes within hydrogels is based on the physical entrapment of the enzymes within the polymer fibre network and does typically not involve chemical or adsorptive interactions (Mohamad et al., 2015; Zucca et al., 2016). Thus, the pore size of the polymer network is the main factor influencing the retention of the enzyme within the hydrogel. The minimum diameter of AaEst2, as calculated from its molecular weight of 34 kDa (Manco et al., 1998), is 4.3 nm (Erickson, 2009), although the effective hydrodynamic diameter can be assumed to be substantially larger. Still, AaEst2 is considerably smaller than the typically reported pore sizes of agarose hydrogels (e.g., (201 ± 36)nm at 5% agarose, determined by atomic force microscopy (Maaloum et al., 1998)) making an effective and permanent retention of AaEst2 by physical entrapment in standard agarose implausible. However, the actual pore size of agarose hydrogels depends strongly on a number of factors like gelation speed (Kusukawa et al., 1999; Labropoulos et al., 2002) and expression organism, as agarose can be extracted from a variety of algae (Selby and Whistler, 1993). In the present study, the probably most relevant factor is that a chemically modified low-melt hydroxyethyl agarose was used which typically forms smaller pores, depending on the degree of substitution (Cook, 1982). The hydroxyethyl substitution and the lack of non-gelling components in low-melt agarose are likely reasons for the observed low amount of leached enzyme compared to agar-based hydrogels. A previous study has shown that AaEst2 is washed out from hydrogels of 3% (w/v) low-melt agarose over longer time periods when employed in perfused microreactors (Maier et al., 2018). As the use of low-melt agarose has the disadvantage of limited applicability at high temperatures, alternatives with both high enzyme retention and temperature stability may be desirable. Agarose derivatives that can be crosslinked for higher temperature stability or that offer reaction sites for covalent attachment are potential candidates for this objective (Zucca et al., 2016).

#### 3.3.2 Enzymatic activity in hydrogels

**FIGURE 10:**
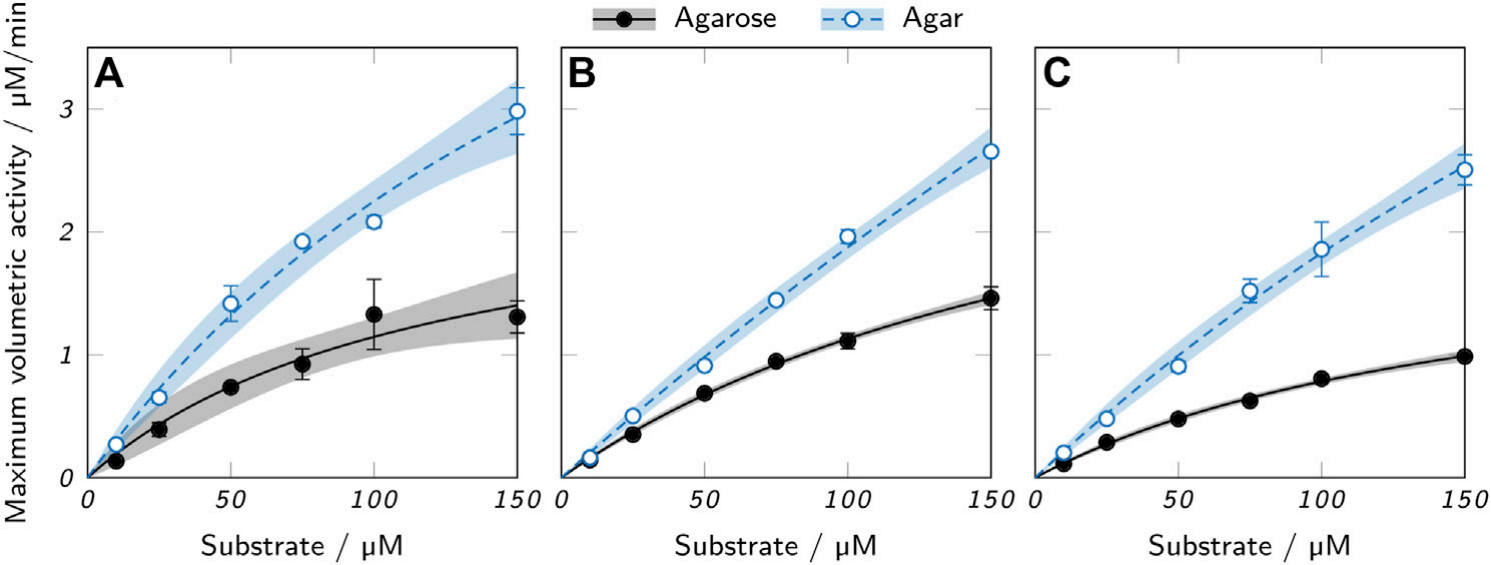
Activity assays of printed low-melt agarose and agar cylinders containing 100 nM AaEst2 and **(A)** 3%, **(B)** 4.5% and **(C)** 6% polymer. The data points represent mean values ± standard deviation (*n* = 3) and were fitted with a Michaelis-Menten equation. 95 % confidence intervals are displayed as shaded areas.

Activity assays with hollow cylinders containing 100 nM AaEst2 were performed in order to compare the maximum enzymatic activity in different hydrogels. Figure 10 shows the results for agarose and agar at three different polymer concentrations and six different substrate concentrations. The resulting kinetics resemble the form of a Michaelis-Menten equation and were fitted accordingly. For both polymer types, there was a slight trend of reduced activity with increasing polymer concentration, as shown in a trend analysis in the supplementary material. This trend is attributable to the reduced diffusibility and hence higher mass transfer limitation of hydrogels with increased polymer concentration, as presented in Figure 8 and discussed above. Across all polymer concentrations, the measured maximum activity of agar hydrogels was roughly twice as high as the activity of the respective agarose samples. Here again, the higher diffusibility of agar hydrogels plays a role, as it allows a higher enzymatic activity due to higher mass transfer rates. However, the more important aspect is most likely the leaching of enzyme from agar hydrogels (Figure 9). The leached enzyme can accumulate in the supernatant and catalyze the reaction without the mass transfer limitations of the hydrogel, resulting in a higher activity of the sample. A quantification of the two effects is not possible with the available data, as the supernatant was only sampled after an incubation time of 2 h. Other possible, but probably minor influences include different surface areas and cylinder heights of the hydrogel samples caused by printing irregularities. Variable printing temperatures are unlikely to cause differences in activity, as AaEst2 is thermostable at 70°C for at least several hours (Manco et al., 1998). At first glance, the results of the activity assays imply agar hydrogels to be the superior material due to the enhanced activity. However, taking enzyme leaching as the most probable reason for the obtained results into account, the enhanced activity is merely a symptom of the unsuitability of agar as an immobilization matrix for AaEst2. Applied in a perfusable reactor, these hydrogels would lose enzyme and hence catalytic activity quickly, failing to provide reusability which is the main advantage of enzyme immobilization. This shows that results of batch experiments cannot be considered alone, but have to be evaluated in the context of additional factors like enzyme leaching.

### 3.4 Further considerations

Besides the already analyzed and discussed aspects like diffusibility, leaching and enzymatic activity, several additional factors may be worth considering when choosing an appropriate ink.

The different viscosities of inks with varying polymer concentration do not only affect the printability, but can influence the handling and processability of these inks during preparation. Low-viscosity inks with a low gelling point (e.g. 3% agarose) can be easily transferred between different containers like the mixing vessel and the printing cartridge, even with pipettes. The handling of highly viscous inks with a high gelling point (e.g. 6% agar) is more difficult and there may be a significant loss of material during preparation which is problematic when working with costly or scarcely available enzymes.

In previous studies, cartridges with enzyme-laden low-melt agarose-hydrogels were prepared for stock and reliquefied as needed (Maier et al., 2018; Peng et al., 2019). This approach allows a high flexibility of the printing process and rapid, on-demand production of enzymatically active hydrogel structures. The high temperatures required to reliquefy agar hydrogels would inactivate most enzymes, making this approach inapplicable in combination with agar-based inks.

For the operation of biocatalytic reactors, the working temperature is a crucial parameter to achieve high activities. The temperature stability of agar hydrogels allows higher working temperatures than could be achieved with low-melt agarose hydrogels. For AaEst2 with its temperature optimum of 70°C (Manco et al., 1998), only agar hydrogels allow an operation at ideal conditions.

All discussed advantages and disadvantages of hydrogels made from low-melt agarose and agar are qualitatively summarized in Table 2.

**TABLE 2:** Qualitative ratings of low-melt agarose and agar bioinks regarding a range of criteria determining their suitability for the printing of biocatalytically active hydrogels.

	Agarose			Agar		
	3%	4.5%	6%	3%	4.5%	6%
Printability	−	0	+	−	0	+
Mechanical stability	− −	−	+	−	+	+ +
Avoidance of leaching	+ +	+ +	+ +	− −	− −	− −
Enzymatic activity	0	0	0	+ ^a^	+ ^a^	+ ^a^
Handling and processability	+ +	+	−	0	−	− −
Reliquefaction with enzyme	+	+	+	− −	− −	− −
Temperature stability of hydrogel	−	−	−	+	+	+

^a^ Determined enzymatic activity of agar hydrogels was increased due to enzyme leaching.

## 4 Conclusion and outlook

The present study compares bioinks with regard to their suitability for the extrusion-based 3D printing of enzymatically active hydrogels. Inks with different concentrations of either low-melt agarose or agar were investigated. A customized printer setup including a heatable nozzle and a cooled substrate was established. Compared to previous publications employing 3% agarose inks and a nozzle without thermal control (Maier et al., 2018; Peng et al., 2019), the newly established setup allowed cleaner and more defined prints without uncontrolled pre-gelation and nozzle clogging. The gel and melting points and the flow behavior of all inks were analyzed using rheological methods. Based on this, suitable printing parameters were determined individually. All bioinks were found to be sufficiently printable to create lattices without overhangs and a height of at least 3 mm with 300 µm layers, but the printing quality was strongly enhanced at 4.5% polymer or more.

The produced hydrogels were characterized regarding mechanical strength and diffusibility. For both properties, a correlation with polymer concentration was observed with highly concentrated hydrogels being more stable and less diffusible. Agar hydrogels were found to be more stable and allow higher diffusion rates of 5(6)-carboxyfluorescein than comparable agarose hydrogels. Enzyme leaching was identified as a major drawback of agar hydrogels, while hardly any leaching from the agarose hydrogels was detected. The leached enzyme is most probably the dominant cause for the observed superiority of agar hydrogels in the performed batch activity assays. This indicates the limited suitability of agar hydrogels for perfused biocatalytic reactors, as the enzyme would be washed out over time. The activity assays showed a small effect of polymer concentration on enzymatic activity.

In summary, agarose inks with at least 4.5% polymer were found to be the most suitable of the investigated inks for the printing of biocatalytic reactors with AaEst2 due to their superior printability and leaching behavior. Drawbacks of the low-melt agarose hydrogels are limited mechanical and thermal stability, not allowing the operation of a reactor at the optimum temperature of AaEst2 which is above the melting point of the tested low-melt agarose.

The observed limitations of the inks could be addressed by a systematic optimization of the ink formulation. Rheological additives could enhance process robustness and printability and enable the manufacturing of more sophisticated geometries including overhangs. Quantitative fidelity measurements should be applied to assess and compare the printability of modified inks. Functionalization of the agarose with polymerizable groups or adding additional monomers would allow a post-curing step that may increase mechanical strength, reduce leaching even further and enable reactions at higher temperatures by enhancing the melting point. Such modifications should be finely tuned to minimize potential enzymatic inactivation by free radicals. Blends of low-melt agarose with agar or unmodified agarose could be used to create inks with defined melting and gelling properties. In order to improve the reaction conditions, systematic screenings of pH, ionic strength and temperature could be performed based on the presented activity assay method.

## Data Availability

The raw data supporting the conclusion of this article will be made available by the authors, without undue reservation.
