# Supplementary material for: Systematic evaluation of agarose- and agar-based bioinks for extrusion-based bioprinting of enzymatically active hydrogels
Source: Front Bioeng Biotechnol. 2022 Nov 21;10:928878. doi: 10.3389/fbioe.2022.928878 (PMC9720278; doi:10.3389/fbioe.2022.928878)
Supplement: Supplementary file 1 [file DataSheet1.PDF]

Supplementary Material

1 MATERIALS AND METHODS

1.1 Mass exchange surface estimation

The potential available surface for the mass exchange of printed cylinders with the surrounding liquid was estimated assuming an ideal hollow cylinder as depicted in Figure S1. For the calculation, certain assumptions are given by the material properties and production strategy. The material density ρ was approximately identical for all hydrogel compositions, the cylinder mass m was kept constant by adjusting the extrusion pressure to yield the desired target weight and the radius r of the cylinders was defined using the Gesim software. The volume of the hollow cylinders V can be calculated from ρ and m.

$$\begin{aligned} \rho &= 1.0\,\mathrm{g/cm^3} \\ m &= 0.1\,\mathrm{g} \\ V &= \frac{m}{\rho} = 0.1\,\mathrm{cm^3} \\ r &= 5\,\mathrm{mm} \end{aligned}$$

Height measurements showed a maximum deviation of 0.4 mm between the mean height h of cylinders printed from different materials:

$$\begin{aligned} h_{min} &= 3.0\,\mathrm{mm} \\ h_{max} &= 3.4\,\mathrm{mm} \end{aligned}$$

- $h_{min} \quad = 3.0\,\mathrm{mm}$
- $h_{max} \quad = 3.4\,\mathrm{mm}$

The base area A_{base} of the cylinders is given by:

$$A_{base} = \pi \left(r_o^2 - r_i^2\right) \tag{S1}$$

The inner radius r_i and the outer radius r_o can be defined as functions of the mean radius r and the thickness of the cylinder d:

$$r_i = r - \frac{d}{2} \tag{S2}$$

$$r_o = r + \frac{d}{2} \tag{S3}$$

With S1, S2 and S3, A_{base} is given as

$$\begin{aligned} A_{base} &= \pi \left(\left(r + \frac{d}{2}\right)^2 - \left(r - \frac{d}{2}\right)^2\right) \\ &= 2\pi r d \end{aligned} \tag{S4}$$

With S4, the volume of the cylinders V is given by:

$$V = A_{base} h = 2\pi r d h \tag{S5}$$

Consequently, d is given as:

$$d = \frac{V}{2\pi r h} \tag{S6}$$

The area available for mass transfer with the surrounding liquid $A_{exchange}$ can be defined as:

$$\begin{aligned} A_{exchange} &= A_{base} + 2\pi r_i h + 2\pi r_o h \\ &= 2\pi r(d + 2h) \end{aligned} \tag{S7}$$

From S6 and S7 follows:

$$\begin{aligned} A_{exchange} &= 2\pi r \left(\frac{V}{2\pi r h} + 2h\right) \\ &= \frac{V}{h} + 4\pi r h \end{aligned} \tag{S8}$$

The maximum and minimum exchange surface area $A_{exchange,max}$ and $A_{exchange,min}$ can be calculated from h_{max} and h_{min}:

$$\begin{aligned} A_{exchange,max} &= \frac{V}{h_{max}} + 4\pi r h_{max} \\ &= 2.00\,\mathrm{cm^3} \end{aligned} \tag{S9}$$

$$\begin{aligned} A_{exchange,min} &= \frac{V}{h_{min}} + 4\pi r h_{min} \\ &= 1.84\,\mathrm{cm^3} \end{aligned} \tag{S10}$$

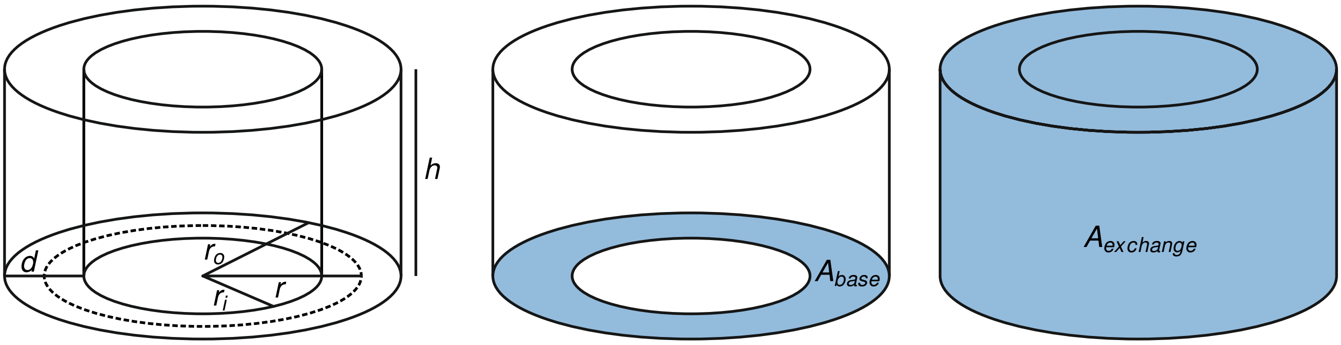

Figure S1: Sketches of hollow cylinders indicating the dimensions used for the mass exchange surface area estimation.

resulting in a difference of $8.7\,\%$ between $A_{exchange,max}$ and $A_{exchange,min}$.

1.2 Estimation of residence time in heat-controlled nozzle

The residence time of ink within the heat-controlled nozzle was calculated to evaluate whether it is sufficient to reach the desired final printing temperature. The printing distance per cylinder l_{print} is given by

$$l_{print} = n_{layers} * U_{cylinder} \tag{S11}$$
$$= n_{layers} * 2 * \pi * r_{cylinder} \tag{S12}$$
$$= 314\,\mathrm{mm} \tag{S13}$$

with the number of layers $n_{layers} = 10$ and a cylinder radius $r_{cylinder} = 5\,\mathrm{mm}$. Neglecting pauses between layers, the maximum printing duration $t_{print,max}$ and minimum printing duration $t_{print,min}$ for one cylinder are dependent on the printing speed ($v_{max} = 7\,\mathrm{mm/s}$ and $v_{min} = 5\,\mathrm{mm/s}$):

$$t_{print,max} = \frac{l_{print}}{v_{min}} = 62.8\,\mathrm{s} \tag{S14}$$
$$t_{print,min} = \frac{l_{print}}{v_{max}} = 44.9\,\mathrm{s} \tag{S15}$$

The volume of the nozzle V_{nozzle} is given by its length $l_{nozzle} = 25\,\mathrm{mm}$ and inner radius $r_{nozzle} = 0.175\,\mathrm{mm}$:

$$V_{nozzle} = \pi * r_{nozzle}^2 * l_{nozzle} \tag{S16}$$
$$= 2.4\,\mathrm{mm}^3 \tag{S17}$$

Finally, the residence time of ink inside the nozzle $t_{residence}$ is given by:

$$t_{residence,max} = \frac{t_{print,max} * V_{nozzle}}{V_{ink}} = 1.5\,\mathrm{s} \tag{S18}$$

$$t_{residence,min} = \frac{t_{print,min} * V_{nozzle}}{V_{ink}} = 1.1\,\mathrm{s} \tag{S19}$$

with $V_{ink} = 100\,\mathrm{mm}^3$ which is given by the target mass of one cylinder $m_{cylinder} = 100\,\mathrm{mg}$ under the assumption that the density of the ink is $\rho_{ink} = 1\,\mathrm{g/mL}$.

1.3 Trend analysis for activity assays with printed hydrogel cylinders

To investigate whether there was a correlation between polymer concentration of the hydrogels and the enzymatic activity determined in batch experiments, all acquired data points were divided by the substrate concentration and plotted over the polymer concentration, as shown in Figure S2. The data points were fitted with a linear fit and the coefficient of determination R^2 was calculated.

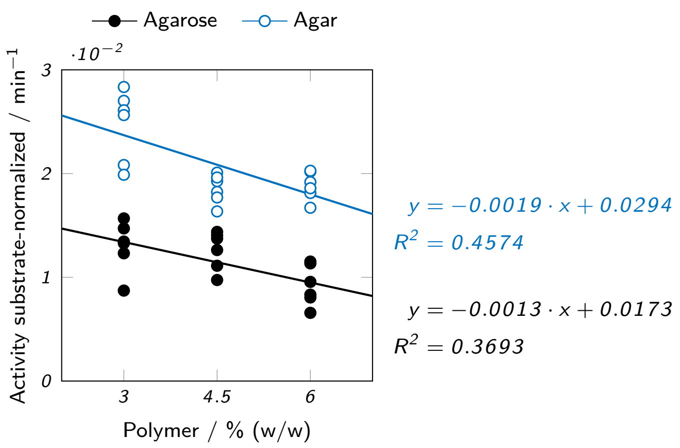

Figure S2: Trend analysis for activity assays of printed hydrogel cylinders made from low-melt agarose- and agar-based inks.
